# Strengthening Linkages Between Public Health and Health Care in Nebraska

**DOI:** 10.5888/pcd16.180600

**Published:** 2019-08-01

**Authors:** Xiaoting Sun, David Palm, Brandon Grimm, Li-Wu Chen

**Affiliations:** 1 Department of Health Services Research and Administration, College of Public Health, University of Nebraska Medical Center, Omaha, Nebraska; 2 Department of Health Promotion, College of Public Health, University of Nebraska Medical Center, Omaha, Nebraska

## Abstract

**Introduction:**

Effective collaboration between public health and the health care system is essential for connecting medical and community health–related resources and improving population health. We investigated the linkages between local health departments and primary care clinics in Nebraska.

**Methods:**

We conducted a mixed-method study by using semistructured in-person and telephone interviews and surveys in 2017 and 2018 with directors of 19 Nebraska local health departments. Interviews and surveys assessed activities and programs that health departments implemented or planned with clinics in their jurisdictions. Barriers, benefits, and opportunities for building the linkages were identified.

**Results:**

Strong linkages existed between local health departments and primary care clinics. Linkages focused on the control and prevention of chronic diseases and on traditional public health programs, including screening for cancer and other chronic diseases, vaccinations, worksite wellness programs, home visits, clinic and medication assistance referrals, health message development, electronic health records data analyses, staff education, and improvements in policies and procedures. The most frequently reported barrier was funding, and the most frequently reported benefit was patient behavior change. The opportunity most frequently reported was chronic disease health coaching.

**Conclusion:**

Extensive linkages exist between Nebraska local health departments and the health care systems in their areas. Additional funding, effective workforce management, community needs assessments, and program evaluation can support joint initiatives to address community health priorities.

**Summary**

**What is already known about this topic?**

Various linkage initiatives between public health and health care systems have been implemented throughout the United States.

**What is added by this report?**

This study was the first to investigate Nebraska’s recent experiences in building linkages between public health and primary care in 2017 and 2018 from the viewpoint of local health departments.

**What are the implications for public health practice?**

The linkage programs and activities and their effect and the barriers, benefits, and opportunities for building linkages identified in this study can be used to shape stronger and broader local and national practices for future collaborations and system integration.

## Introduction

The health care system is undergoing dramatic changes, from volume-based reimbursement to value-based reimbursement, to deal with the challenge of managing population health (1). One change is the emergence of new health care delivery models such as accountable care organizations and patient-centered medical homes (2,3). These models have strong financial incentives to reduce costs while improving the quality of care and health outcomes through better care coordination (4). To achieve these goals for both individuals and populations, public health practitioners and health care providers must eliminate the cultural divide that exists between them and form effective partnerships that connect medical and community resources.

Opportunities are increasing for public health agencies to work closely with the health care system by building linkages and collaborations. The Institute of Medicine’s 2012 report explored promising integration models for the 2 systems, described the degree of integration, and developed principles for successful integration (5). Public health practitioners and health care providers have worked together to integrate their systems and health objectives (6). A 2013 nationwide survey found that 84% of state public health practitioners engaged in collaboration activities but that these activities were primarily client-oriented and focused on secondary rather than primary prevention (7). A 2016 study found that in communities that implemented population health activities involving multisector partners, resident death rates from cardiovascular disease, diabetes, and influenza were reduced (8). A recent report concluded that these partnerships are critical components of a comprehensive community wellness approach that will ensure seamless care and prevention for everyone (9). Linkages not only improve individual health, but also work upstream to address the policies and environmental and social factors that influence community and population health (10).

Nebraska ranked tenth among all states in America’s Health Rankings in 2014 and thirteenth in 2017 (11). In public health funding, Nebraska ranked twentieth in 2017 with an expenditure of $95 per person (11). Nevertheless, in some health areas, such as obesity, infectious diseases, and disparity in health status, Nebraska was far below national averages and needed collaboration between health care and public health. We assessed linkage activities between local health departments and primary care clinics in Nebraska in 2017 and 2018.

## Methods

We conducted a mixed-method study by using semistructured in-person and telephone interviews and surveys to assess linkage programs and activities between local health departments and primary care clinics. We collected data in 3 steps. First, we conducted in-person and telephone interviews with 19 Nebraska health department directors in 2017. The interview consisted of 12 semistructured questions on linkage activities that were planned or implemented with the clinics in the health department’s jurisdiction. All interviews were recorded and transcribed, and common themes were identified. In July 2018 we administered a 19-question survey to assess the progress and effects of the departments’ linkage activities (Table). Respondents could choose from answers provided that were based on information obtained from the 2017 telephone interviews, give other answers, or fill in the numbers or rating scores. The number of each given answer selected and the weighted rating scores were calculated, and the numbers were added. In August and September 2018 we conducted another round of interviews with 5 health department directors to gain more in-depth knowledge.

**Table:** Responses to Survey of Nebraska Local Health Departments (N = 16), 2018

Survey Question	No. of LHDs Responding	Answer^a^
**1. If you are involved in screening for prediabetes, please estimate the total number of clients screened by the department.**	14	**—**
Number screened (received an HbA_1C _blood test)		217
Number referred to physician clinics		546
Number referred by physician clinics		146
**2. What source(s) of clients are referred into the pre-diabetes program?**	14	—
Physicians clinic		10
Federally qualified health center		8
Hospital		7
Health department programs		9
Other community organizations		7
Worksite wellness program		8
Self-referral		11
Other		5
**3. If you are involved in hypertension screening, please estimate the number of clients.**	15	**—**
Number screened		2,637
Number referred to physician clinics		459
**4. How have you been involved in promoting cancer screening? Please check all that apply.**	16	**— **
Traditional media (newspapers, television, radio)		13
Social media (Facebook, Twitter)		15
Health fairs		15
Posters		9
Health coaching		16
Other (please specify)		5
**5. If you issue fecal occult blood test kits, how many kits have been issued and how many people have been referred to a physician for follow-up?**	16	—
Number of kits analyzed		1,792
Number of abnormal readings		34
Number of people referred to primary care clinics		34
**6. If you have a worksite wellness program, what activities are provided? Please check all that apply.**	12	—
Health risk appraisal assessment		11
Screening (eg, for diabetes, hypertension, cholesterol)		11
Health education for high risk behaviors		12
Number that referred workers to primary care clinics		7
Technical assistance for policy changes		7
**7. How many community health workers do you employ?**	16	**— **
1		4
2		5
3		3
≥4		3
None		1
**8. If you employ community health workers, what are their functions and activities? Please check all that apply.**	15	—
Health coaching		12
Translation and interpretation		9
Screening (eg, for diabetes, hypertension, cholesterol)		10
Assist clients in enrolling in Medicaid or exchange plans		4
Medication management assistance		3
Home visits		4
Connect clients to medical and community services		14
Work closely with care coordinators or other staff members of patient-centered medical homes		4
Other (please specify)		6
**9. If you provide a full range of vaccinations for children and influenza vaccinations for adults, is there a strong partnership with primary care clinics in your district to minimize gaps in coverage?**	13	**—**
Yes		8
No		5
**10. Are you involved in the following programs or activities with primary care clinics? Please check all that apply.**	15	—
Home visitation programs for children		3
Connect low-income clients with medication assistance programs		12
Develop educational messages that are used by and prepared for physician clinics		7
Review clinic materials for literacy standards		4
Provide education to clinic staff members about emerging and re-emerging diseases		3
Assist clinics in analyzing data from electronic health records		4
Assist clinics in coordinating behavioral health services		1
Build relationships between care team extenders (eg, pharmacists) and clinics		4
Assist in developing quality improvement policies and procedures		10
Assist in developing referral procedures for community services (eg, health coaching)		12
Other (please specify)		9
**11. What are your new opportunities for linkages with primary care clinics in your district?**	16	**—**
Mental health/substance abuse		10
Dental health services		8
Development of evidence-based policies		11
Lead screening		12
Chronic disease health coaching		14
Prevention of opioid abuse		9
Other (please specify)		2
**12. How would you rate the level of integration with the primary care clinics in your county or district? Please rate for each clinic using 4-level scale^b^.**	15	—
Clinic no. 1		2.7
Clinic no. 2		2.6
Clinic no. 3		2.3
Clinic no. 4		2.4
Clinic no. 5		2.5
Clinic no. 6		2.6
**13. Do you have any formal agreements (eg, contract, memorandum of understanding) on linkage projects with one or more physician clinics?**	16	—
Yes		10
No		6
**14. What are the barriers that you face when working with clinics?**	16	—
Clinic capacity		10
Lack of vision		9
Administrative (LHD)		1
Administrative capacity		11
EHR status/EHR vendor support		11
Funding		14
Other (please specify)		5
**15. What are the benefits your LHD gains by working with primary care clinics?**	16	—
An increase in referrals to your evidence-based community programs		10
Better health outcomes		11
Closing care loops		10
Increased collaboration with community-based physician extenders		9
Reduced duplication of services		7
Reinforcement of messages to patients for behavior change		14
Other (please specify)		4
**16. How will linkage projects most likely be funded in the future? Please rank the options from 1 to 4 with 1 the most likely and 4 the least likely.**	16	—
Grant funds		3.4
Medicaid funds		2.3
Private insurer funds		2.1
Revenue-generated program funds		2.1
**17. How do you rate your level of involvement in helping the nonprofit hospitals in your district to develop their Community Health Needs Assessment and Implementation Plan? Please rate for each hospital using a 4-level scale^c^.**	16	—
Hospital no. 1		1.7
Hospital no. 2		1.8
Hospital no. 3		1.7
Hospital no. 4		2.2
Hospital no. 5		2.3
**18. How do the priorities in your Community Health Improvement Plan compare with the priorities in the Community Health Needs Assessment of nonprofit hospitals in your area?**	16	—
Priorities are the same or almost identical		14
About half of the priorities are the same		2
Most priorities are different		0
**19. How closely matched are your implementation efforts with the nonprofit hospitals in your district?**	16	—
Closely matched and cohesive		7
Somewhat matched but not cohesive		8
Not closely matched		1

Abbreviations: —, not applicable; EHR, electronic health record; LHD, local health department.

a Values are counts of health departments who selected that answer, total number, or weighted score depending on question types. The data were collected for the period from July 1, 2017, to June 30, 2018.

b Rating scale was 1 to 4: 1 = mutual awareness (clinic and health department informed about each other’s activities), 2 = cooperation (sharing of some resource), collaboration (joint planning and execution), 3 = partnership (closely working on program level), 4 = partnership (close working relationship on a programmatic level; user perceives no separation). All health departments in Nebraska worked with 6 or fewer clinics during study period, and scales were weighted.

c Rating scale was 1 to 4: 1 = limited (consult) or no involvement, 2 = some involvement (provided data and helped data analysis, 3 = a member of the planning committee), and 4 = extensive involvement (prepared all or a large portion of the plan and helped shape the priorities). All health departments in Nebraska worked with 5 or fewer nonprofit hospitals during study period, and scales were weighted.

## Results

Eighteen of 19 health department directors completed the first-round interview, and 16 responded to the survey. Responses showed that in addition to traditional programs (eg, tracking communicable and food-borne illness outbreaks, emergency preparedness, environmental health programs) several strong linkages with primary care clinics already existed in 5 areas we identified: the National Diabetes Prevention Program, screening services, worksite wellness programs, vaccination services, and other programs and activities. Ten health departments had formal agreements on linkage projects with clinics. Most linkage activities focused on control and prevention of chronic diseases. Because of limited resources and large geographic coverage areas, most health departments worked with only a few clinics.

### Collaboration between health departments and clinics

**National Diabetes Prevention Program**. Fourteen of the 16 health departments that responded participated in one common linkage program, the evidence-based National Diabetes Prevention Program, which involves lifestyle improvement for patients with prediabetes through healthy eating, increasing physical activity, controlling stress, and losing weight. From July 1, 2017, through June 30, 2018, these 14 health departments screened 217 clients for prediabetes and referred 546 at-risk clients to primary care clinics (Table). Of the 14 health departments, 11 selected patients from self-referral, 10 from primary care clinics, 9 from health department programs, 8 from federally qualified health centers, 8 from worksite wellness programs, 7 from hospitals, and 7 from community organizations. Each health department employed a nurse or a community health worker to serve as the health coach for the 16-week program. Although most directors felt the program was successful, many said that it worked most efficiently when patients were referred into the program by primary care clinics and when patients’ results from the program were shared with the clinics. Without patient referrals from clinics, health departments often struggled to enroll enough patients.

**Screening services**. Fifteen health departments offered screenings for diabetes, hypertension, and cholesterol at multiple community sites and worksites, and at the health department. Participants with abnormal readings were referred to clinics. All 16 were involved in promoting breast, cervical, and colorectal cancer screenings through multiple routes, including health coaching. Fifteen promoted screenings in social media campaigns, 15 in health fairs, 13 through traditional media, and 9 by using posters. Five health departments reported other methods, such as paying for transportation for low-income women or distributing free fecal occult blood test kits for colorectal cancer screening. Through screening services, health departments identified at-risk clients, referred them for follow-up with a health care provider, and assisted them in navigating the health care system. Cancer screening rates increased in both rural and urban areas in Nebraska from 2017 to 2018. However, sometimes health care providers sent little or no information back to health departments.

**Worksite wellness programs**. Twelve health departments reported providing worksite wellness programs. Although activities varied, all programs offered health education for high-risk behaviors (eg, tobacco use, alcohol use, obesity), and 11 health departments administered a health risk appraisal survey to identify worksite employee health needs and developed an action plan to address the needs. Eleven health department worksite programs provided screening for diabetes, hypertension, and cholesterol; 7 referred people to primary care clinics; and 7 provided technical assistance in developing health-related policies at worksites, such as offering nutritious options in company vending machines.

Health departments collaborated with community partners in developing worksite wellness programs. For example, in one rural community, after the health department conducted the health risk appraisal survey, the department partnered with a large employer, a physician clinic, and a hospital to develop a comprehensive wellness plan. During the implementation of the plan, the health department provided health promotion and education materials and resources, the clinic conducted screenings and follow-up consultation, and the hospital assessed occupational health risks.

**Vaccination services**. Eight of 13 health departments that offered vaccination services reported close partnerships with clinics for providing vaccinations. Because about half of the health departments provided a full range of vaccinations for children and influenza vaccinations for adults, clinics sometimes referred their patients to health departments for vaccinations. Increased vaccination rates were observed from 2017 to 2018. One common challenge was documenting the number of children who received appropriate vaccinations, because some clinics failed to report the numbers to the Nebraska State Immunization Information Registry and because of the incompatibility between the registry and the clinics’ electronic health record systems.

**Other programs and activities**. Fifteen health departments also reported other activities, either working with clinics or targeting people in their community. Twelve educated clinic staff members about lead testing, emerging diseases (eg, Ebola, Zika virus), and re-emerging diseases (eg, tuberculosis). Twelve health departments assisted clinics in developing referral procedures for health-related community services. Nine departments helped clinics analyze electronic health records data to identify high-risk patients and encourage referrals into health department programs. Moreover, health departments worked with clinics on conveying health messages. Seven departments developed educational materials that could be placed in physician offices. One sent out tip-of-the-month messages with clinic logos. Another department reviewed educational materials from clinics to ensure that they met health literacy standards. Eight departments helped clinics build relationships with care team extenders, such as pharmacists. Ten assisted clinics in developing quality improvement policies and procedures. To help high-risk clients, 3 health departments conducted home visits for children aged 3 years or younger and provided education to mothers about nutrition and breastfeeding. Five departments connected low-income clients with medication assistance programs to lower their drug costs.

### Barriers to collaboration

The most significant barrier to building linkages that the 16 health departments surveyed reported was funding (14 departments), followed by administrative capacity in clinics (11 departments), compatibility of electronic health record systems (11 departments), clinic capacity (10 departments), and lack of vision (9 departments). Other barriers mentioned were limited health department capacity, public health not being physician-centric, and not having the same strategic priorities.

A major barrier mentioned was the cultural divide between health care providers and public health professionals. Public health workers emphasize disease prevention and focus on factors influencing health outcomes (eg, health behaviors, social determinants of health), whereas clinicians focus on treatment.

Another major barrier was the lack of capacity to connect health care and public health systems. Sharing information in a timely manner was sometimes difficult because of technology and workforce problems. Some health department did not have electronic health record systems that were compatible with those in clinics, and some departments did not have a workforce competent in information exchange. Many rural areas had shortages of both physicians and public health professionals, which made it more difficult to develop and maintain strong linkages. Fifteen of the 16 health departments used at least one community health worker to serve as a bridge. The common functions for these workers included connecting patients with medical and community services (14 departments), providing health education (12 departments), conducting chronic disease screenings (10 departments), and language translation (9 departments). Some workers also assisted patients in enrolling in Medicaid or exchange plans (4 departments), conducted home visits (4 departments), worked closely with care coordinators or other staff members of patient-centered medical homes (4 departments), and assisted in patient medication adherence (3 departments).

Another capacity issue was the lack of funding. Besides Medicaid and private insurers, linkage projects were and will likely continue to be funded by grants from the Nebraska Department of Health and Human Services. Most primary care clinics also rely on grants to build expertise in data analysis and care coordination. However, neither health departments nor clinics had any assurance that these funds would continue at the same level. The lack of sustainable funding produced substantial variability in programs and limited their reach. Some linkage projects were operating in only a few clinics in the local health department’s district.

### Attitudes and perceived opportunities

All 16 health departments recognized the benefits of working with clinics. Reinforcement of messages to patients for behavior change was identified by 14 departments, followed by better health outcomes (11 departments), increased referrals to their evidence-based community programs (10 departments), closing care loops (10 departments), increased collaboration with community-based physician extenders (9 departments), and reduced duplication of services (7 departments). Other benefits included filling gaps in vaccination for children, addressing community priorities, and ensuring evidence-based policies.

All 16 health departments also identified many new linkage opportunities. Fourteen departments identified opportunities to collaborate with clinics on chronic disease health coaching, 12 on lead screening, 11 on the development of evidence-based policies, 10 on mental health and substance abuse, 9 on prevention of opioid abuse, and 8 on dental health services. Some departments had already explored activities in mental and dental health. For example, one department screened children in schools for mental health. Some were working with clinics to ensure that patients with mental conditions made regular visits and adhered to medication regimens. Some departments organized training in the Mental Health First Aid program, a national certification program to teach skills for responding to the signs of mental illness and substance use. Because dental health was a priority need in most rural areas, and the number of patients visiting hospital emergency departments for dental issues increased, some departments provided various community dental preventive services (eg, fluoride varnish, dental sealants) to fill the gaps and reduce unnecessary dental expense. 

## Discussion

Our study showed that strong and varied linkages existed between health departments and primary care clinics. Programs such as the National Diabetes Prevention Program, worksite wellness programs, screening services, and vaccinations were provided by all health departments. Most departments also worked with clinics by providing education to clinic staff members, assisting in developing referral procedures, building relationships between clinics and care team extenders, developing educational messages, and assisting in quality improvement and data analysis of electronic health records. Only a few health departments engaged in activities such as medication adherence and assistance or home health visits.

Though linkages varied by type and range of activities, there was potential to build on past experiences and explore new opportunities. Health departments should pursue multiple funding options to build sustainable partnerships. One option is mandatory community benefit spending by nonprofit hospitals. A national investigation showed that spending by tax-exempt hospitals on community health improvement initiatives was inadequate and sometimes unrelated to community health needs (12), which was also echoed by a study conducted in Nebraska (13). Hence, if health departments partnered with nonprofit hospitals and hospitals spent more on community initiatives, additional funds would be available for linkage programs. Other options include generating revenue through donations or third-party reimbursement. By assisting clinics to participate in federal programs, such as the Chronic Care Management Program and the Medicare Pre-Diabetes Program, health departments could share the additional revenue from these programs (14).

To overcome barriers such as incompatibility of electronic health record systems and lack of a skilled workforce, more investment is needed in health information technology and workforce training. Some health departments initiated educational programs for medical students and residents. They could also partner with colleges of public health to train primary care providers in competencies of working under an integrated system. In some areas, community health workers can assist clinics’ care coordinators to track high-risk patients who missed appointments and work with pharmacists to assist in medication adherence.

Collaboration activities between Nebraska health departments and primary care clinics were similar to those in other states. One study interviewed 40 public health and primary care practitioners from 4 states in 2014 and 2015 and classified barriers for collaboration into 3 types: institutional barriers (stressful work environments in clinics, different motivations from collaborating groups, billing issues, and isolated systems and jurisdictions), process-related barriers (lack of shared knowledge and understanding, poor and inconsistent communication, and inability to share data because of multiple data platforms), and resource-related barriers (shrinking resources, lack of shared strategic planning and priorities to address community needs, and lack of program sustainability) (15). These findings highlighted the urgent need for system and structural changes.

The Institute of Medicine identified 5 key levers for building an effective collaborative model based on a multistate, multipartner quality improvement collaborative on hypertension control (16). The levers were leadership at the local, state, and national levels; identification of community and clinical resources; having multiple data sources; having standardized protocols; and pursuing multiple financing options. To address barriers to collaboration, the institute suggested using a quality improvement approach, establishing a public health workforce skilled in health system transformation, and using strategic planning to identify resources. Briss (17) summarized several practical linkage experiences, such as incorporating risk behaviors assessment into clinic workflows by programming questions into the electronic health records systems and generating automatic reminders for the assessment. A Netherlands study in 7 neighborhoods described a stepwise approach to develop integrated district plans and promote collaboration between public health and primary care at the local level by using 2 central tools — district health profiles and policy dialogue (18). The key was to involve appropriate collaborators in dialogue (eg, general practitioners, residents) and invest sufficiently in sharing aims and data from stakeholders.

Given the shift from volume-based to value-based reimbursement, more clinics will likely adopt the patient-centered medical homes model or join accountable care organizations. The new models provide strong incentives to improve care coordination and population health outcomes. The changes in reimbursement and delivery models provide strong incentives to focus on population health and collaborate with local health departments and community organizations. As the goals of clinics and health departments become more similar, more research will be needed to identify the most effective models of collaboration and the programs and activities that produce the greatest health improvements.

Strong linkage projects that focused on prevention were between local health departments and primary care clinics in Nebraska. Although many projects were successful, some were concentrated in a few communities and clinics. As more clinics become patient-centered medical homes or join accountable care organizations, these linkage projects should grow. The major challenges are to expand the health department workforce and find new funding options to support linkage programs. Finally, once these linkage programs are implemented, it is critical to evaluate their impact. Colleges of public health and other academic institutions can play a useful role in this evaluation process.
